# Developing an active implementation model for a chronic disease management program

**DOI:** 10.5334/ijic.994

**Published:** 2013-06-17

**Authors:** Margrethe Smidth, Morten Bondo Christensen, Frede Olesen, Peter Vedsted

**Affiliations:** The Research Unit for General Practice Aarhus and The Section for General Medicine, Institute of Public Health, Bartholins Allé 2, Aarhus University, 8000 Aarhus C, Denmark; The Research Unit for General Practice Aarhus, Institute of Public Health, Bartholins Allé 2, Aarhus University, 8000 Aarhus C, Denmark; The Research Unit for General Practice Aarhus, Institute of Public Health, Bartholins Allé 2, Aarhus University, 8000 Aarhus C, Denmark; The Research Unit for General Practice Aarhus, Institute of Public Health, Bartholins Allé 2, Aarhus University, 8000 Aarhus C, Denmark

**Keywords:** implementation, disease management, Denmark, Chronic Care Model, Breakthrough Series, PaTPlot

## Abstract

**Background:**

Introduction and diffusion of new disease management programs in healthcare is usually slow, but active theory-driven implementation seems to outperform other implementation strategies. However, we have only scarce evidence on the feasibility and real effect of such strategies in complex primary care settings where municipalities, general practitioners and hospitals should work together. The Central Denmark Region recently implemented a disease management program for chronic obstructive pulmonary disease (COPD) which presented an opportunity to test an active implementation model against the usual implementation model. The aim of the present paper is to describe the development of an active implementation model using the Medical Research Council’s model for complex interventions and the Chronic Care Model.

**Methods:**

We used the Medical Research Council’s five-stage model for developing complex interventions to design an implementation model for a disease management program for COPD. First, literature on implementing change in general practice was scrutinised and empirical knowledge was assessed for suitability. In phase I, the intervention was developed; and in phases II and III, it was tested in a block- and cluster-randomised study. In phase IV, we evaluated the feasibility for others to use our active implementation model.

**Results:**

The Chronic Care Model was identified as a model for designing efficient implementation elements. These elements were combined into a multifaceted intervention, and a timeline for the trial in a randomised study was decided upon in accordance with the five stages in the Medical Research Council’s model; this was captured in a PaTPlot, which allowed us to focus on the structure and the timing of the intervention. The implementation strategies identified as efficient were use of the Breakthrough Series, academic detailing, provision of patient material and meetings between providers. The active implementation model was tested in a randomised trial (results reported elsewhere).

**Conclusion:**

The combination of the theoretical model for complex interventions and the Chronic Care Model and the chosen specific implementation strategies proved feasible for a practice-based active implementation model for a chronic-disease-management-program for COPD. Using the Medical Research Council’s model added transparency to the design phase which further facilitated the process of implementing the program.

Trial registration: http://www.clinicaltrials.gov/(NCT01228708).

## Introduction

The number of people living with chronic conditions is growing with the rising life-expectancy, improved treatment options and growing diagnostic activity and because of inappropriate lifestyle. Meeting the needs for care for people living with multimorbidity or even with a single chronic disease is challenging for society. Healthcare related and social benefit costs for people with chronic obstructive pulmonary disease (COPD) are high and much may be gained from better coordination of care for this patient group in Denmark both in terms improved care and fewer overall costs [1]. Like in many other countries, there is room for improvement in the multidisciplinary effort [2], and new ways of managing chronicity need to be tested [3]. The quality of care for patients with COPD may be improved by adopting a more systematic and structured approach to the care [4, 5] and by enhancing patients’ ability to better cater for their own needs and manage their chronic condition [6, 7]. The provision of healthcare to people with multimorbidity must be guided both by societal cost-efficiency considerations and by consideration of the disease and its management for patients and healthcare professionals alike [8]. It is essential to design an efficient healthcare strategy towards this group of patients to ensure that they are offered seamless, professional, effective treatment in hospitals, in general practices and in the municipalities [9–11]. Comprehensive healthcare provision may be furthered within a framework that motivates the different stakeholders in the healthcare system to work together [12–14].

According to Wagner et al., improvements within parts of the healthcare system may facilitate system reform in which informed, activated patients interact with prepared, proactive healthcare teams. They developed the framework called ‘the Chronic Care Model’ in which patients are given the right care at the right place at the right time to optimise the use of resources [15]. Within this model, the care is evidence-based, planned and proactive, and the model is currently being used with success in several healthcare systems [16–18]. The model aims to improve the results of the care for the whole population and it involves a planned and coordinated effort between general practitioners, hospitals and groups working at the local or municipal level to further health-promotion and rehabilitation [19].

COPD is an under-diagnosed, irreversible and potentially life-threatening condition where secondary prevention, treatment and rehabilitation can help control the symptoms, increase the patient’s quality of life and delay disease progression [20]. Newly published results indicate that among people aged 35 years or older, 14.3% are living with COPD in Denmark [21]; and of these only approximately 28% have been diagnosed [22].

**Figure 1.**
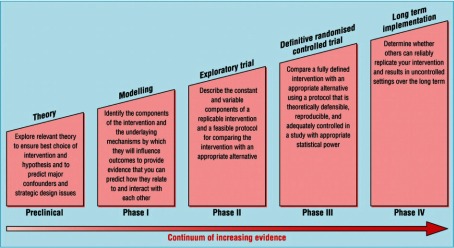
The suggested phases for design of a complex healthcare intervention, developed by the Medical Research Council, UK. Campbell et al., 2000 [28].

A new disease management program for COPD inspired by the Chronic Care Model was implemented in 2008 in The Central Denmark Region [23]. The program was based on The Danish College of General Practitioners’ Guideline and the World Health Organisation’s GOLD Guidelines [24]. However, targeted or planned efforts are often not specified when new guidelines are being implemented; it may therefore take a very long time before these changes, if any, actually occur where implementation processes are passive [25, 26]. To stimulate active implementation, we introduced a planning model for the development of a complex intervention as the active implementation is. The model is a framework that was developed by Michelle Campbell et al. for The British Medical Research Council (Figure 1). This model explicates the process of developing a complex intervention and it sheds light on each of the components involved in the intervention [27, 28]. To visualise this and the process timeline, Perera et al. developed the PaTPlot [29] which we applied in the present study (Appendix 1). We wanted to change the way general practitioners and their staff worked and interacted with the municipality and the hospitals to provide a more comprehensive and population-based care than the one presently offered where there is no shared responsibility for patients. We wanted to measure the change at patient level. The usual practice of disease management program implementation was used for comparison with the active implementation as one of the arms in a randomised trial [23]. To control for an overall trend, a comparable, neighbouring municipality’s general practices and patients formed an external control group.

### Aim

The aim of this paper is to describe the development of a model for active implementation of a disease management program, which was based on the Chronic Care Model, using the British Medical Research Council’s ‘Framework for design and evaluation of complex interventions to improve health’ to develop the complex intervention model. We first developed the model and then tested its use in a randomised controlled trial. The results are reported elsewhere.

## Methods

### Setting and structure

The healthcare system in Denmark is tax-financed and citizens have free access to most healthcare services at the point of care. The healthcare system has a list-based gatekeeper system, and 99% of all Danish citizens are listed at a general practice. Many preventive and rehabilitation issues are dealt with by the municipalities which typical have 40–50,000 inhabitants. There are five regions in Denmark, each with some one million inhabitants [30]. The regions are responsible for provision of general practice and for provision of secondary care; citizens may be admitted to regional hospitals for treatment and specialised procedures [31].

Each of the approximately 3600 Danish general practitioners has on average 1600 patients on their lists. The general practitioners act as gatekeepers towards the rest of the healthcare system; where access requires referral from a general practitioner, except for emergency admission, a few specialist treatments (eye and ENT) and rehabilitation in the municipalities. The general practitioners are independent contractors with the region and are currently remunerated on a combination of fee-for-service and capitation basis (75/25) [32].

The study period spanned from November 2009 to December 2011. The study was conducted in the western part of The Central Denmark Region where the three regional hospitals, Ringkoebing, Herning and Holstebro provided secondary care. Two comparable municipalities in the area provided the setting for the present study. The randomised trial with an intervention and a control group was conducted in Ringkoebing-Skjern municipality which had almost 58,000 inhabitants and 38 general practitioners organised in 15 practices; we added an extra, external control group which was Ikast-Brande municipality with close to 40,000 inhabitants and 25 general practitioners in 12 practices. All practices had staff that undertook part of the consultation or conducted consultations on their own. The staff were employed by the general practitioners and were mainly educated as nurses, laboratory technician or secretaries.

### Approach and methods

As an independent body that encourages and supports research with the aim of maintaining and improving human health, the Medical Research Council in the UK has developed a framework [33] for developing and evaluating complex interventions in randomised controlled trials. The Medical Research Council suggests a sequenced process that starts with exploration of relevant theory. This phase is followed by four separate phases for development and evaluation (see Figure 1). New guidance on using the framework was published in 2008. These new guidelines suggested adapting the intervention to local circumstances and that although understanding the process is important it does not replace evaluation of the outcomes [34].

Phase I: Identification of evidence base and intervention components; modelling of outcome and process; identification of any incentives or barriers to intervention components geared to modify patients’ or health professionals’ behaviour [35].

Phase II: Feasibility and piloting of components, processes and procedures identified in Phase I; adaptation and modification to ensure optimal effect.

Phase III: Evaluation of fit-for-purpose and assessment of the effectiveness and efficiency of the intervention [36].

Phase IV: Surveillance and monitoring of the intervention including, among others, its rate of uptake; long-term follow-up.

We used this framework to develop a model for an active implementation strategy for a disease management program for COPD.

The Medical Research Council’s framework states that preliminary work is essential to identify and establish the active components of the intervention, to ensure that they can be delivered effectively during the trial, and to identify explicit success criteria [33]. First we explored the theoretical evidence base for the implementation components; then we used focus groups with patients and health professionals to assess the active components we had identified, and we conducted a randomised trial to demonstrate their effect; we thus joined two of the phases, namely II and III [33]. We wanted to design an intervention in conformity with current research findings, which recommend that chronic disease management programs should consist of at least one healthcare professional-directed intervention, one organisational intervention and one patient-related intervention to support self-management [37].

The present paper reports the application and interpretation of each phase. The result of the RCT is reported elsewhere.

### Ethics

The study was recommended by the Committee for Multicentre Studies of the Danish College of General Practitioners and the Association of Danish General Practitioners (MPU 17-2009) and it was approved by the Danish Data Protection Agency (J. nr. 2008-41-2855), the Danish National Board of Health (J. nr.: 7-604-04-2/71/EHE); the RCT is indexed at http://www.clinicaltrials.gov/(NCT01228708).

## Results

Below we report the separate results for the Medical Research Council’s framework Phases I–IV.

### Phase I

We aimed to change the way general practitioners and their staff worked and interacted with the municipality and the hospitals to optimise the care and we wanted to measure the change at patient level. To stimulate the process, we chose to use a locally accepted opinion leader to introduce and support the implementation both in the municipality and in the general practices [38].

In the preclinical phase, the Chronic Care Model was identified as a suitable model for designing efficient implementation elements, and we decided to build our active intervention on the following core dimensions in the model: policies and resources, self-management support, delivery system design, decision support, organisation of the healthcare and clinical information system [11, 39]. Interventions that contain at least one Chronic Care Model element have been shown to improve clinical outcomes and processes of care and, to a lesser extent, the quality of life for patients with chronic illnesses [40].

We searched MEDLINE for systematic reviews and papers on implementing change in general practice for shared care for patients with chronic conditions. A snowball-search identified further literature. We did not conduct a systematic review ourselves. Using the current literature and empirical knowledge, we sought inspiration from interventions that had successfully and efficiently implemented new initiatives in primary care [26]. Two reviews of different types of continued professional education showed that studies using interactive methods were generally more effective in changing general practitioners performance and in improving patient care than traditional lectures [41, 42], and another review found that people who develop the guidelines must consider the implementations process and not only the elements in the guideline [43].

**Figure 2.**
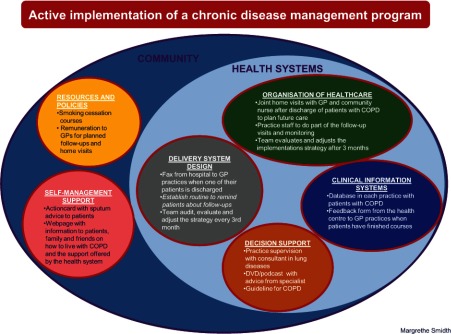
A model for an active implementation of a disease management program. Implementation components organised within the Chronic Care Model’s core dimensions.

The general practices in the intervention group were invited to participate in an introduction session where the local opinion leader together with the project leader introduced the project. This introduction was followed by three two-and-a-half-hour sessions over seven months. Figure 2 displays the interacting components based on the Chronic Care Model and the below section offers a detailed description of our use of these components.

### Implementation elements

### Community

### Policies and resources

We negotiated our implementation strategy with the municipality of Ringkoebing-Skjern, which actively supported implementation of the strategy by increasing the number of free smoking cessation courses from two to eight and the number of self-management courses from one to five during the study period. General practitioners could then refer more patients with COPD to the municipality care scheme. The stronger commitment shown at the municipal level and from the general practitioners also made it possible to launch initiatives that enhanced patients’ ability to cope with their disease in general and to better manage exacerbations [44].

At the municipality’s health centre, the format of the self-management courses was one that took an appreciatory approach with dialogue between the patient and the health professional about the patient’s range of choices and opportunities, available treatment options and the patient’s readiness to change habits. In Ringkoebing-Skjern, an experienced patient, i.e., a person who had COPD and had come to terms with how to function in daily life with a chronic disease, participated in the courses together with health professionals. The courses were designed to offer the patients the possibility of obtaining more insight into the care of their disease and to enhance their confidence and competence in terms of self-management [45, 46]. Patients could contact the municipality’s health care centre on their own initiative or they could be referred by their general practitioner to the centre for one one-hour screening session to explore and balance expectations, wishes and motivations for change. The subsequent course consisted of sessions twice a week for ten weeks and follow-up sessions after one, three and nine months. The contents of the courses varied according to the patients’ needs and aimed to give them more insight into the illness pathology, enhance their knowledge about physical training, instruct them in breathing exercises, create opportunities for social networking, provide support for smoking cessation, support knowledge and insight into their own psychological and physical situation, discuss and provide new inspiration for sexual life and clarify medication issues. Each session included one hour of physical training and discussion of nutrition while preparing a shared healthy lunch. Emphasis was given to participatory activities with dialogue-based knowledge exchange to aid development of competences to act.

We negotiated with the relevant regional authorities to expand an existing agreement for paying for joint home visits to citizens aged 75+ who had recently been discharged. Under the extended agreement, general practitioners were reimbursed for home visits to all patients irrespective of age who had been hospitalised because of their COPD and who were discharged and needed follow-up [47]. The agreement involved a shared care approach where the general practitioner and the community nurse employed by the municipality met with the patient and planned future care which aimed to raise both the patient’s and the health professionals’ sense of a united, coherent and proactive healthcare system [48, 49].

### Self-management support

We developed targeted self-management support by designing an action card with sputum advice for patients based on the research by Robert Stockley [50, 51]. We obtained permission to use the card from the group in the UK which had used it in one primary care trust and had shown that the use of the advice given on the action card reduced the number of acute hospital admissions [52]. We expanded the card by using the green-yellow-red action plan recommended in the Chronic Care Model (Appendix 2) [53]. The expansion consisted in adding written advice on how to behave in case of a worsening of the symptoms of cough, breathlessness and/or sputum production.

We wanted to inspire and encourage family, friends and patients to talk openly about the disease by providing disease-specific knowledge and therefore developed a webpage with information about the following issues: COPD; the support, help and aid provided by the municipality; local support groups and the general practices. Parts of the website were targeted to health professionals. These parts introduced best practice and the newest evidence for treatment of COPD, which was also taught at the educational meetings [54]. Furthermore, the website included information about the randomised study and the questionnaires used both for patients and general practitioners.

### Health system

### Delivery system design

Part of the intervention consisted of enhanced information sent to the general practitioners about newly discharged patients with COPD. We introduced a system whereby the hospital department informed general practices immediately by fax when one of their patients who had been admitted with COPD was discharged. Thus, the general practice could contact the community nurses to plan a joined visit if needed [55].

We also encouraged the general practices to establish a routine for inviting patients to attend preventive follow-up visits annually or more frequently if specified by the program. General practitioners were remunerated with a special fee for such follow-up visits [23], but the organisation of the follow-up visits was left at the discretion of the individual general practices. Some had already established routines for inviting patients with other chronic diseases for follow-up and wanted to use the same system for patients with COPD. Others followed our advice to invite patients during the month of their birthday and others liked and used the idea of inviting 1/12 of the alphabetically listed patients with COPD each month [56–58].

For the purpose of continued quality improvement, the practice team was advised to audit, evaluate and adjust the strategy every third month [56]. Some practices already had regular meetings where they discussed their routines and others introduced continued evaluations of their strategy at meetings with all health professionals present.

### Organisation of healthcare

The general practices were encouraged to organise their routines of delegating work so that practice staff did part of the follow-up visits and the monitoring of patients with COPD in order to make use of each health professional’s unique skills and knowledge and to optimise the time used by the general practitioners [56]. Several practices designed written manuals to avoid repeating tests or procedures.

### Decision support

We wanted to enable easy access to up-to-date evidence and local advice from specialists in lung diseases and therefore created podcasts for consultations with smoking cessation, instruction in the use of spirometry and a follow-up consultation and made them available on the website [54].

Summarising initiatives to improve general practitioners’ information management, Grimshaw et al. describe that the use of a local specialist or opinion leader may have some effect where a change in behaviour is sought [59]. We therefore arranged for extra access to expert knowledge by allowing general practices to draw on a local consultant in lung diseases both for advice and for practice consultations.

### Clinical information system

Better disease control demands precise identification of the population in question [56]. We therefore provided each general practice with a list of their patients with COPD. The patients were identified by a previously developed COPD algorithm [60].

The general practitioners expressed that they would like to have feedback from the municipality’s health centres when a patient with COPD had finished a course at the centres. A form for this purpose was developed in collaboration between the staff at the health centres, the patients, the general practitioners and the research group. This exchange of information most often enhanced the respect for and knowledge of each other’s professions and improved the patients’ feeling of being cared for within the context of a united healthcare system [61–63].

### Implementation process

As a framework for the three educational sessions with the general practices, we used the Breakthrough Series [64–67], which the Chronic Care Model suggests may be used to introduce planned, targeted changes. During the educational sessions, the practice teams worked in groups first with the general practitioners and the staff in the same group and next with the general practitioners and the staff in separate groups. Each practice developed a plan detailing which elements they wanted to implement in their own practice and how and when these elements were to be implemented. The practices had a choice between some elements, whereas other elements were mandatory. We used experienced facilitators and experts to change routines in general practices, to provide updates on secondary care, to inform of the municipality’s courses and to present the patients’ views recently shown to work to the satisfaction of general practitioners and staff [68, 69].

We adopted the strategy of academic detailing to induce changes in general practitioners’ behaviour. This strategy has previously been shown to be efficient for teaching general practitioners how to report adverse drug effects, even if it was most successful in the first four months after the intervention [70]. Other trials have found that the strategy works better in smaller than in larger practices [71]. A Cochrane review found a small, but consistent change when the strategy was used in face-to-face meetings [72]; in a paper discussing this Cochrane review, Vedsted and Lous argue that academic detailing can be used when monitored closely [73]. A previous Danish study also suggested that practice visits are an effective method for identifying incentives and barriers to change general practitioners’ clinical behaviour [74].

The implementation process therefore involved outgoing visits to every intervention practice to, first, explore and/or address any challenges encountered and, second, to assist the general practice in the implementation of its individual goals. Clinically educated and well-experienced in furthering change in general practice, the project leader assumed the role of implementation facilitator. During the intervention period, the implementation facilitator monthly rang one designated person from each practice to follow-up on changes suggested at the visits and to get a briefing on the progress made. E-mails with information on the overall progress of the project were sent to each of the general practitioners.

To introduce the project at each of the three hospitals involved, the project leader held meetings with the staff involved where the roles of the hospital were explained and any questions clarified. The person appointed responsible for faxing the general practices with information on COPD patients discharged during the study period was regularly contacted by phone.

The municipality health centre staff was introduced to the project at staff meetings. During the full study period, the project leader regularly visited the municipality’s health centre to discuss the progress of the project in general and the centre’s communication with the general practices in particular. The community nurses were informed about the project at a special presentation.

The contact and follow-up was done by one person so that all information was centralised. This approach was chosen to enhance a continuous and coherent implementation process.

### Phase II: Explorative trial

We created our active implementation model as a complex intervention that combined the individual components presented above into a multifaceted whole. The inclusion of multiple elements meant that the intervention could be tailored to the individual’s needs which, overall, increased the likelihood that change was accomplished. Hence, the intervention was designed in line with Grimshaw et al.’s review of more than 235 multifaceted trials, which showed that combined interventions are more effective than single interventions [75, 76].

A dialogue-centred approach was adopted [77]. We therefore formed focus groups (one with health professionals and three with patients) and consulted these groups to elicit their views both during the development stage and when deciding which elements to include in the model. The interview guide was developed with open-ended questions containing topics identified from literature and empirical knowledge to be important for integrated care. The project leader guided the discussion, and notes were taken by the local health care facility manager. Both afterwards discussed the notes and agreed on the essential themes. Quotes from recurrent themes in the focus groups and the consultations are presented in Table 1.

Minor adjustments were made after the input sessions with the stakeholders. For example the feedback form became more detailed and patients whose general practitioners were in the intervention group were sent a flyer about the study and possible places for seeking information about the disease. The same information was presented on a poster in the intervention general practices.

We used the PaTPlot to ensure that every step of the study with all its different components was described in detail and to ensure due timing of the implementation process [29]. The PaTPlot enabled us to depict the two arms in the block and cluster-randomised trial and the external group in a graphical and standardised manner for easy interpretation and clear comparison between the three arms (see Appendix 2).

### Phase III: Definitive randomised controlled trial

A national disease management program for COPD [78] produced in conformity with the principles of the WHO GOLD guidelines [79] and a clinical guideline from The Danish College of General Practitioners [80] was the background material for the Central Denmark Region’s own disease management program [23]. The usual practice of disease management program implementation was used for comparison with the active implementation as one of the arms in the randomised trial [23]. To control for an overall trend, a comparable, neighbouring municipality’s general practices and patients formed an external control group. We used patient questionnaire surveys and registry data before and after the intervention. The randomised study and the effect on outcome measures are reported in separate papers.

### Phase IV: Long-term implementation

We used components that are directly implementable and that may easily be used on a continuous basis in the general practices as well as in the municipality’s health centre and at the hospitals. It will therefore be possible to adapt and generalise the used methods and elements in a way that can sustain and further develop the effect of the intervention.

## Discussion

We developed an active implementation model for a chronic disease management program for patients with COPD using the step-wise approach for development of complex interventions described by the Medical Research Council. We incorporated Wagner’s Chronic Care Model together with knowledge on multifaceted processes for implementation including the Breakthrough Series, and we found the method applicable for our implementation purpose and suitable for a randomised controlled trial. We expected that the results from the trial will have an effect on health care utilisation and on the patients’ evaluation of their care similar to the findings of a review of both qualitative and quantitative studies [42]. We also expected that the beneficial effect would be more pronounced if the study had continued for more than one year [81].

We adhered to the current recommendations [37] that active implementation should consist of multiple elements each targeting different areas and actors. We used theory and established components to develop a multifaceted or complex intervention in the pre-clinical phase that covered the areas identified in the Chronic Care Model as components instrumental in producing a change in healthcare settings where informed, activated patients interact with prepared, proactive practice teams [9, 10, 15].

Using the Breakthrough Series within the context of a randomised trial allowed the general practices to constantly adjust and evaluate the components in the model. Such continual improvement is essential for disease management program implementation in an interactive setting of a healthcare system where three partners—hospital, municipality and general practitioners—are involved, not least in the long-term.

### Strengths and limitations of the study

Using the transparent and rigorous approach of the Medical Research Council’s framework together with the Chronic Care Model, the Breakthrough Series and the PaTPlot strengthened the development of the active implementation and improved its reproducibility. We were able to integrate suggestions from focus group interviews with patients and from individual interviews with health professionals to adjust the implementation model. The local wishes were then addressed and integrated into the model before the randomised controlled trial was performed. The involvement of stakeholders in the development of phase II gave them ownership and is believed to have motivated adherence to the active implementation of the program.

We might have obtained even more suggestions and valuable input to the active implementation by trying it out in general practices before conducting the randomised controlled trial. Such suggestions and input may, however, have been influenced by the type and size of the pilot general practices. A representative pilot study from which to adjust the active implementation would have required a longer study period and would not have been feasible from an economic perspective. We allowed for adjustments along the way to meet local needs and therefore found it valuable to use qualitative methods to localise the intervention as also recently suggested by the Medical Research Council [34]. The focus groups added knowledge of health professionals’ and patients’ prioritisation of the need for care from the healthcare system and how they wanted it delivered. The health professionals’ discussion and comments to the questionnaire improved the questions and added valuable input regarding the sequence of the questions. The action card was developed further following the patients’ advice. We used the qualitative approach to guide us and might have been just as informed by just having one patient and one health professional focus group and then saved some time.

Many studies have been published since 2005 using the framework suggested by the Medical Research Council and they all seem to interpret the contents and purpose of the development phases differently. There seems to be little agreement on the key tasks involved in the development of complex interventions as stated, among others, by Maindal in their description of the development of a complex health education [82]. We found the use of the PaTPlot most helpful as it illustrates both the contents of the intervention and the timeline. Many have experienced the challenges of implementing disease management programs [43, 83]. Most advise policymakers to address the following issues: local adaptations and joint development of care, sharing of data, empowering of patients, many stakeholders working together and preventive measures to avoid the chronic diseases [84, 85]; similar recommendations were made in a diabetes improvement initiative [86].

A huge disadvantage of this complex intervention model is that it gave us no opportunity to test the impact of the single components. Discussions and research is ongoing on whether single or multifaceted interventions have the biggest impact on change [75]. We have chosen a multicomponent intervention to be able to address the different areas considered important to change the delivery of healthcare. Reviews have shown that integrated care programs have positive effects on the quality of care [19]. In a Dutch study [48, 87], national guidelines for general practice were implemented by post and by post with outreach visitors. The Dutch study found both more extensive use of the guidelines and higher awareness of their existence whichever intervention was used. We therefore expected that the general practices would choose the components they found most relevant. In future research, one could consider to focus on different combinations of components and on differently sequenced implementations. Offering the general practices a range of components to choose between in a catalogue of possibilities instead of using a fixed intervention may have strengthened the general practices’ ownership and encouraged greater commitment to the implementation.

Other future research could study the impact of one person having all the contact to the stakeholders during the study period. Such an approach may be instrumental in accomplishing some changes, but it might also have blocked others as personality preferences might have influenced compliance with the suggestions discussed at practice meetings.

The active implementation was developed for COPD, but with minor disease-specific adjustments it may be used for other chronic diseases. It will be possible for others to follow the PaTPlot and replicate this active implementation and reproduce the intervention.

We have developed the model for an active implementation as a complex intervention and it will be evaluated by patients using the tool Patients Assessing their Chronic Illness Care (PACIC) and by health care utilisation reported in other papers [Patient-experienced effect of an active implementation of a disease management program for COPD—a randomised trial, submitted to BMC Family Practice] [The effect of an active implementation of a disease management program for chronic obstructive pulmonary disease on healthcare utilisation—A cluster-randomised controlled trial, submitted to BMC Health Services Research]. We could have used the Assessment of Chronic Illness Care (ACIC) tool, but then we would have needed to translate and validate it into Danish first. This tool might have enabled us to track if the change was sustainable over time [88], though one trial using the ACIC did find that they would need a more sensitive measurement tool to measure any changes when implementing the Chronic Care Model [89].

## Conclusions and implications

Using the step-wise procedure recommended by the Medical Research Council, we developed an active implementation model for a disease management program where we used COPD as a model disease. We expect the systematic description of each component in the complex intervention to facilitate implementation of disease management programs and to ease further research on the effects hereof. We have trialled the model in a small-scale randomised, controlled study whose results will be reported separately. After this, we expect that the active implementation model for management of COPD can be used for implementing change in the management of other chronic diseases, but we recommend further research into organisational and disease-specific challenges and into the effect of different implementation and improvement efforts in the short and long-term run.

## Authors’ contributions

MS, PV, MBC and FO participated in the study and the drafting of the manuscript. PV and MS developed the design and MS coordinated the study and drafted the manuscript. MS carried out the focus group interviews and consultation with local general practitioners and health professionals. All authors have read and approved the final manuscript.

## Acknowledgements

The study was carried out at The Research Unit for General Practice in Aarhus and further funded by Ringkoebing-Skjern Municipality, the Central Denmark Region and The Medical Research Fund at Aarhus University, Denmark.

We are grateful to patients and health professionals in Ringkoebing-Skjern Municipality who have contributed to the development of this active implementation model. We thank Dr. Erik Juul Jensen, Hospitalsenheden Vest, The Central Denmark Region for providing teaching and support from the hospitals. Susanne Rystok, Sundhedscenter Vest, Ringkoebing-Skjern proofread the part about the municipality’s courses for patients with COPD and is kindly thanked. We thank Lars Foged (GP), Ulla Svendsen (RN, Director of Health Centre Vest, Ringkoebing-Skjern municipality) and Steen Vestergaard-Madsen (Chief consultant at the Central Denmark Region) for participating in the development of the overall study.

## Reviewers

**Roberto Nuño Solinis**, Director of O+berri, Basque Institute for Healthcare Innovation, Sondika (Bizkaia), Spain

**Michaela Schiøtz**, MSc Public Health Science, PhD, Scientist, Steno Health Promotion Center, Steno Diabetes Center, Denmark

**Bert Vrijhoef**, Professor of Health Systems and Policy, Saw Swee Hock School of Public Health, National University of Singapore & Professor of Chronic Care, Scientific Center for Care and Welfare, Tilburg University, The Netherlands

## Figures and Tables

**Table 1.** Quotes from three focus groups with patients and one with general practices and several consultations with the health centre staff

Patients	General practices	Municipality’s Health Centre staff
“I would like one system for accessing my file both for the hospital, the health centre, my doctor and myself or at least a system where there is exchange between those who treat me when something has happened to or with me”	“We really need a report on what has happened when the patient has been at the health centre”	“We want patients from all social groups to feel equally comfortable while attending classes”
“My doctor should know about and inform me about the COPD classes at the health centre and the groups for people with my condition”	“Please, update us on the newest and most current medication advice”	
“I would like to know my lung function and what it means that it is reduced”		
“I would like to be able to handle more—like medication—myself”		

## Appendix

**Appendix 1**. The PaTPlot depicting the timeline and the contents. Squares illustrate fixed objects, e.g., printed materials like questionnaires. Circles illustrate that an activity was involved in the component, e.g., Continued Medical Education meetings.

## Appendix 2

**Action card**. Advice for patients based on the research by Robert Stockley on the one side. On the other side, advice on what to do if the breathlessness, the cough or the sputum get worse; green is as normal, yellow is when it is more and the patient can step up his or her medication to the maximum dose, use a PEP flute and do controlled breathing exercises. If the breathlessness, cough or sputum get a lot worse than normal, it is in the red column and the patient needs to take a maximum dose of medication, do breathing exercises, start on prednisolon and must contact a doctor.
